# Are we nearly there yet? Coverage and compliance of mass drug administration for lymphatic filariasis elimination

**DOI:** 10.1093/trstmh/tru204

**Published:** 2015-01-08

**Authors:** Neal D. E. Alexander

**Affiliations:** MRC Tropical Epidemiology Group, Department of Infectious Disease Epidemiology, Faculty of Epidemiology and Population Health, London School of Hygiene and Tropical Medicine, Keppel Street, London, WC1E 7HT, UK

**Keywords:** Elimination, Lymphatic filariasis, Mass drug administration

## Abstract

Lymphatic filariasis has been targeted for elimination by 2020, and a threshold of 65% coverage of mass drug administration (MDA) has been adopted by the Global Programme to Eliminate Lymphatic Filariasis (GPELF). A recent review by Babu and Babu of 36 studies of MDA for lymphatic filariasis in India found that coverage, defined as receipt of tablets, ranged from 48.8 to 98.8%, while compliance, defined as actual ingestion of tablets, was 22% lower on average. Moreover, the denominator for these coverage figures is the eligible, rather than total, population. By contrast, the 65% threshold, in the original modelling study, refers to ingestion of tablets in the total population. This corresponds to GPELF's use of ‘epidemiological drug coverage’ as a trigger for the Transmission Assessment Surveys (TAS), which indicate whether to proceed to post-MDA surveillance. The existence of less strict definitions of ‘coverage’ should not lead to premature TAS that could impair MDA's sustainability.

In 1997, the World Health Assembly called for lymphatic filariasis to be eliminated as a public health problem. WHO's response included the launch of the Global Programme to Eliminate Lymphatic Filariasis (GPELF) in 2000. An important tool for elimination is mass drug administration (MDA) with albendazole combined with either ivermectin or diethylcarbamazine citrate (DEC), for which the minimum effective coverage of the total population is considered to be 65%.^1^ Published in 2010, this GPELF report is subtitled ‘halfway towards eliminating lymphatic filariasis’, in reference to the target elimination date of 2020. In 2015, we are three-quarters of the way.

The 65% threshold for effective coverage is based on a modelling study which, for the specific setting of Pondicherry (India), estimated that eight rounds of ivermectin would give 99% probability of elimination.^2^ For combination therapy, this study found that five or six rounds might be sufficient. The GPELF recommends carrying out a Transmission Assessment Survey (TAS) after at least five rounds of MDA, in order to determine whether the area requires further rounds of MDA, or can proceed to the surveillance stage.^3^ The most recent GPELF progress report shows that, of 73 filariasis-endemic countries, 60 have started MDA, of which 15 have since stopped MDA nationwide.^4^

The recent review by Babu and Babu of 36 studies of MDA for lymphatic filariasis in India highlights the divergence of some reported coverage information from the ‘effective coverage’ specified by the GPELF.^5^ Combining urban and rural areas within each study, coverage ranged from 48.8 to 98.8%. India's National Vector Borne Disease Control Programme indicates coverage of MDA (DEC+albendazole) of between 82 and 88%, for the years 2006–2013.^6^ Provisional GPELF data from India in 2013 showed coverage of 71.4% of the target population.^4^

However, coverage is not ‘effective’ if, for example, it includes delivery of drugs that are not subsequently ingested. Babu and Babu noted that the literature distinguishes ‘coverage’ (the proportion of eligible people who received the antifilarial tablets) from ‘compliance’ (the proportion of eligible people who actually ingested the tablets). The distinction would be immaterial if the drugs were almost always ingested, but Babu and Babu found that, on average, the difference between coverage and compliance was 22%.

Defining coverage in terms of the eligible population corresponds to ‘drug coverage’ as defined in the WHO TAS manual.^7^ By contrast, ‘epidemiological drug coverage (programme coverage)’ refers to the total population.^1,7^ Stolk et al.'s coverage of 65% also refers to the total population. They note that the presence of a group who never ingest the MDA drugs, for example due to ineligibility, is an ‘important threat to the effectiveness of mass treatment’. In India, those pregnant, below 2 years of age or seriously ill are not eligible.

Eligible individuals consistently declining MDA also contribute to this threat to effectiveness. In total, 29 of the 36 reviewed studies reported factors associated with low compliance, the most common being fear of side effects, lack of perceived need for the drugs and being away from home when the drugs were delivered to relatives. These are similar to those found in a global review of compliance,^8^ whose five recommendations included tailoring programs to local conditions, minimizing the impact of adverse events and promoting the broader benefits of the MDA program.

To achieve elimination, the required duration, and the level of effective coverage of MDA can be expected to vary between settings, perhaps greatly. One key determinant is likely to be vectorial capacity, which depends on characteristics of the local mosquitoes, such as density and biting rate. Other elimination and eradication programmes are likely to be instructive. For example, the endgame of Guinea worm (dracunculiasis) eradication suggests that the ‘last inch as opposed to the last mile’ will ‘be the most costly and require special efforts’.^9^ Several studies on filariasis MDA highlight the existence of areas refractory to control, often called ‘hotspots’.^10,11^ These studies note the need for flexible control strategies in such areas, in terms of TAS methodology and MDA duration. What should not be flexible are definitions of elimination metrics such as coverage. Decisions on proceeding to transmission assessment (TAS) should be based on GPELF's ‘epidemiological drug coverage’, and subject to a verification survey.^7^ Otherwise, as Babu and Babu note, TAS surveys may be done prematurely. Any additional rounds of MDA would then impose sustained, possibly unexpected, strain on fund-raising and staff morale, which could cause effectiveness to falter.
